# The Substitution Effect of the Fine Aggregate in Concrete with Oyster Shell

**DOI:** 10.3390/ma17246148

**Published:** 2024-12-16

**Authors:** Yao-Ming Hong, Sharan Roy Choudhury

**Affiliations:** 1 Department of Natural Resource and Environmental Studies, National Dong Hwa University, Hualien 97401, Taiwan; 2 Department of Green Technology for Sustainability, Nanhua University, Chiayi 62248, Taiwan; sharanroychoudhury@gmail.com

**Keywords:** oyster shell, concrete, compressive strength, specific gravity

## Abstract

The construction industry contributes significantly to global carbon emissions, accounting for approximately 27% of total emissions. With the increasing demand for concrete, there is a growing need to explore alternative materials that can reduce environmental impact. This study investigates the potential of using oyster shell powder, a waste material, as a partial replacement for fine aggregates in concrete. The methodology involves replacing fine aggregates with oyster shell powder in varying proportions (0%, 10%, 20%, 30%, and 40%) and testing the compressive strength of the resulting concrete after 56 days. The concrete mix used in this study consists of 16.67% cement, 33.33% fine aggregates, and 50% coarse aggregates (10–20 mm). The findings show that increasing oyster shell content reduces the concrete’s compressive strength; however, at 40% replacement, the concrete still achieves a compressive strength of 30 MPa, which meets the required building strength standards. Additionally, the use of oyster shell powder reduces the unit weight of concrete by approximately 10% at the 40% substitution rate, due to the lower specific gravity of oyster shells compared to sand. This research highlights the potential of using oyster shell powder as a viable solution for mitigating oyster shell waste while providing an alternative material for fine aggregates in concrete.

## 1. Introduction

Concrete is a highly consumed material in construction, composed mainly of fine aggregate, coarse aggregate, and cement, which, when combined with water and cured, form a strong and durable structure. Aggregates are inert materials like sand, gravel, or crushed rock, which combine to form a fluid mass that hardens into concrete. Due to its durability and versatility, the global demand for concrete continues to grow. The United Nations Environment Program reports that the industry uses 1.6 billion tons of Portland cement annually, producing 12 billion tons of concrete. On average, 4.7 tons of concrete per person are produced globally each year. Natural resources are heavily consumed in this process, with fine and coarse aggregates accounting for 60% to 85% of the concrete’s volume. Their composition, size, and quality affect the concrete’s durability and strength.

Cement is the most expensive component in concrete, while aggregates provide strength and occupy the largest volume. Quarrying and transport of aggregates lead to environmental issues such as noise, dust, and biodiversity loss [1]. To mitigate resource depletion, researchers are exploring alternative materials like seashells as potential aggregate replacements. Sand mining, another environmental concern, leads to riverbed erosion, water quality degradation, and loss of groundwater. Alternatives to sand, like crushed sands and industrial byproducts such as fly ash, blast furnace slag, and quarry dust, are being explored to minimize environmental impacts [2].

Blast furnace slag and coal ash (fly ash and bottom ash) are promising substitutes. Glass powder, construction waste, and quarry dust also show potential. Seashells, rich in calcium carbonate, are being researched for partial replacement of cement in concrete due to their similarity to limestone [3]. The use of recycled aggregate with a coating of recycled cement paste is another promising technique to enhance concrete’s mechanical behavior and sustainability [4].

In recent years, there has been a growing interest in using alternative materials to replace fine aggregates (FA) in concrete, primarily to address environmental concerns related to the extraction of natural aggregates [5]. Among these alternatives, oyster shells stand out due to their unique properties and sustainable nature. Oyster shells, a byproduct of the seafood industry, are abundant and can be repurposed effectively in concrete production, offering both environmental and material benefits [6]. Unlike other substitutes, such as fly ash or crushed sand, oyster shells contain a significant amount of calcium carbonate (CaCO_3_), which plays a key role in cement hydration, enhancing strength and durability in concrete [7]. Additionally, the use of oyster shells helps mitigate waste disposal issues associated with the seafood industry, promoting a more circular economy [8].

This paper aims to investigate the potential of oyster shells as a sustainable alternative to fine aggregates in concrete. Specifically, this study examines the physical and mechanical properties of concrete mixtures incorporating varying proportions of crushed oyster shell aggregates replacing traditional fine aggregates. By evaluating parameters such as workability, density, and compressive strength, this research seeks to assess the feasibility of using oyster shells in concrete production as a means to reduce the environmental impact of traditional materials. This study also aims to contribute to waste management efforts by exploring the repurposing of oyster shell waste, aligning with circular economy principles and promoting the sustainable use of industrial byproducts in construction.

## 2. Literature Review

The use of oyster shells in the construction industry has been explored for a long time, with multiple studies conducted to assess their potential. Researchers have investigated the effect of oyster shells on concrete, studying their application as both a binding agent substitute and an aggregate substitute. This section reviews findings from various studies to provide an understanding of the current knowledge surrounding the use of oyster shells.

### 2.1. Fine Aggregate in Concrete

Concrete consists of cementitious material and fine and coarse aggregates. Aggregate typically serves as an inert filler, making up 60 to 80 percent of the volume of concrete. Despite being considered an inert filler, aggregate plays a crucial role in defining the elastic characteristics, dimensional stability, and thermal properties of concrete. Aggregates are classified into two types: fine aggregates and coarse aggregates. The quality of aggregate is a key factor in concrete performance, as most aggregates used in concrete are much stronger than other ingredients. The compressive strength of these particles significantly influences the properties of lightweight aggregate concrete.

Before mixing, it is essential to understand the aggregate’s physical properties to achieve a desirable concrete mixture. Important properties include shape and texture, size gradation, moisture content, specific gravity, reactivity, soundness, and bulk unit weight. These properties, along with the water-to-cement ratio, determine concrete’s strength, workability, and durability. Fresh concrete is highly influenced by the aggregate’s shape and texture. Smooth, rounded aggregates tend to increase workability compared to rough, angular, or elongated aggregates [9].

According to ASTM-C33-18 [10], fine aggregates must have no more than 45% passing through any sieve while being retained on the next consecutive sieve, with a fineness modulus between 2.3 and 3.1. For continuous shipments from a given source, the fineness modulus should not vary by more than 0.20 from the base fineness modulus.

### 2.2. Use of Waste Shells in Concrete

Taiwan generates approximately 2,200,000 metric tons of oyster shell waste annually [11]. The Taiwan Sugar Corporation, in collaboration with the Industrial Technology Research Institute, has found a more profitable application: extracting calcium carbonate from the shells to use as a non-active ingredient in pharmaceuticals [12]. This innovative use of oyster shells highlights their potential as a sustainable material, not only in pharmaceutical applications but also in construction, where calcium carbonate’s properties can be leveraged to reduce reliance on traditional cement materials. The use of waste seashells, such as oyster shells, as a partial replacement for cement or aggregate in concrete has been the subject of several studies. Seashells are a readily available and abundant waste material that can potentially be used to reduce the environmental impact of concrete production. Previous research has shown that the inclusion of seashells in concrete can have a positive impact on the mechanical and durability properties of the material. The high calcium carbonate content of seashells can contribute to the formation of calcium silicate hydrate during the hydration process, which can enhance the strength, durability, and resistance to certain types of degradation, such as sulfate attack and chloride penetration, of the concrete. The improved durability of seashell-based concrete can be attributed to the chemical composition of the seashells [13].

Oyster shells are primarily composed of calcium carbonate (CaCO_3_), which, when used as a partial replacement for fine aggregates, could play a role in enhancing the cement hydration process. Calcium carbonate can release calcium ions when mixed with water, potentially aiding in the formation of calcium silicate hydrate (C-S-H), the compound responsible for the strength and durability of concrete. In addition, the high content of calcium carbonate in oyster shells makes them similar to limestone, which is widely known for its influence on cementitious behavior. The presence of oyster shells in the mix could thus contribute to improving the cement matrix, which would enhance the concrete’s overall mechanical properties.

However, the incorporation of seashells in concrete can also present some challenges. Some studies have found that the use of seashells as a partial replacement for cement or aggregate can lead to a decrease in the mechanical properties of the concrete, such as compressive strength and flexural strength. This reduction in mechanical properties may be due to the differences in the physical and chemical properties of seashells compared to traditional cement and aggregates. Seashells may also have a higher water absorption capacity, which can affect the workability and curing of the concrete mixture [14].

To address these challenges, researchers have explored various ways to optimize the use of seashells in concrete, such as investigating different methods of processing the seashells (e.g., grinding, calcination) and exploring the use of seashells in combination with other supplementary cementitious materials, such as fly ash and ground granulated blast furnace slag. Overall, the use of waste seashells in concrete is a promising approach for reducing the environmental impact of concrete production and promoting sustainability in the construction industry. 

The reduction observed in the mechanical properties of concrete may be due to the differences in the physical and chemical properties of seashells compared to traditional cement and aggregates. Seashells may also have a higher water absorption capacity, which can affect the workability and curing of the concrete mixture [15]. Despite these challenges, the use of waste seashells in concrete is an area of active research and development. Researchers are exploring ways to optimize the use of seashells in concrete, such as by investigating the optimal replacement levels, processing methods, and mix design considerations. By incorporating waste seashells in concrete, the construction industry can potentially reduce the environmental impact of concrete production, while also providing a sustainable solution for the disposal of this abundant waste material [16].

The use of periwinkle shells in concrete, both partially and entirely, was explored using five different design mixes [17]. It was concluded that periwinkle shells can be used to produce medium-strength lightweight concrete. However, strength and workability decreased as the shell content increased, with a more significant effect in high aggregate/cement ratio mixes. Partial and full substitution of fine and coarse aggregates with oyster shells was examined, revealing that compressive and flexural strength decreased as the oyster shell content rose [18]. Oyster shell ash was evaluated as a mortar aggregate substitute, with tests showing that compressive strength remained stable, while the influence of organic compounds on hardening and long-term strength enhancement via fly ash was also studied [19]. The interaction between oyster shell and cement paste was assessed, revealing no significant interaction; however, the elastic modulus decreased by 10% with 20% oyster shell substitution [20]. The performance of periwinkle shell lightweight concrete under elevated temperatures was studied, showing that as the water-to-cement ratio and temperature increased, compressive strength and density decreased, with the shells being suitable only for temperatures below 300 °C [21]. Increasing oyster shell substitution was found to negatively impact long-term strength, elastic modulus, and freeze-thaw resistance, although creep and chemical attack tests showed no significant changes [22]. Calcined periwinkle, oyster, and snail shells were tested as admixtures in mortar, with optimal compressive strength observed at 10% periwinkle, 15% oyster shell, and 20% snail shell ash replacement [23]. The use of various seashells as partial cement replacements was investigated, revealing that ground seashells reduced water consumption and improved workability in hot conditions [24]. Coarse aggregate was replaced with Pachymeninx aurita shells in varying amounts, showing that compressive and flexural strength decreased as shell content increased [25]. Granitic chippings were replaced with periwinkle shells in concrete, resulting in a decrease in density and strength, but concluding that periwinkle shells can be used for reinforced concrete, especially in riverine areas [26]. Fine and coarse aggregates were replaced with periwinkle shells, with 30% substitution producing satisfactory results without significantly reducing compressive strength [27]. Mussel and oyster shell ash were examined as partial Portland cement replacements, finding that compressive strength decreased with increased shell content, but mussel ash performed better than oyster shell ash [28]. The peak stress and elastic modulus were observed to increase as the oyster shell substitution ratio rose from 0% to 20%, but showed an exponential decline as the substitution ratio increased further from 20% to 100% [18].

### 2.3. Impact of Shell Substitution on Concrete Workability

Concrete workability refers to how easily freshly mixed concrete can be mixed, poured, solidified, and finished with minimal loss of homogeneity. Workability affects the strength, quality, and appearance of concrete and even associated labor costs during installation and finishing processes [29]. The partial and full use of oyster shells as an aggregate in concrete was studied, testing water–cement ratios of 0.45 and 0.55, and using an air-entraining superplasticizer to enhance workability. The results showed that coarse aggregate replacement reduced the workability of wet concrete as oyster shell content increased. For 50% and 100% aggregate replacement, there was no change in slump height, recording a slump reading of 0 mm. For fine aggregate, workability increased at 30% replacement [22]. The substitution of coarse and fine aggregates with oyster shell was further investigated, testing water–cement ratios of 0.40, 0.45, 0.50, 0.55, and 0.60 for coarse aggregate replacement, and 30% and 50% oyster shell replacement for fine aggregate. The slump value for coarse aggregate rapidly decreased as oyster shell content increased, and when oyster shell replacement exceeded 50%, the slump values approached zero. However, for fine aggregate, the slump value increased as oyster shell content increased at the same water-cement ratio. The increase in slump value was attributed to poor natural fine aggregate availability, which caused a lack of coherence between the cement paste and aggregates, leading to segregation and poor workability. At 50% oyster shell replacement, the slump remained constant, indicating poor grain structure and friction between oyster shell particles, reducing cohesion with the cement paste and causing liquidity issues [30]. Shells from periwinkles, a freshwater snail, were used as an alternative aggregate in concrete, with the study showing that as the percentage of periwinkle shells in the mix increased, the concrete’s compressive and tensile strengths, as well as its workability, decreased [17]. Concrete properties with partial substitution of fine aggregate with oyster shell were studied, testing 5%, 10%, and 20% oyster shell replacement using two types of oyster shell powder with different fineness moduli. Crushed oyster shell reduced the workability of concrete due to its dried, thin plate structure. The use of water-reducing admixtures helped improve workability, but for 20% replacement, the improvement was minimal [20].

The effects of using various seashells as a cement replacement in mortar for masonry and plastering were investigated, with the study finding that increasing the percentage of ground seashells in Portland cement reduced the water requirement and improved setting times, thereby enhancing workability. The replacement of cement with ground seashells also reduced the amount of cementitious material and increased the free water content in the mix. The study noted that oyster shell powder had a higher water requirement compared to other seashells, which was attributed to the larger surface area of the oyster shell particles, necessitating more water to coat the surface [24]. The effects of partially and completely substituting coarse aggregate with *Pachymelania aurita* shells were examined, with the results from five sets of specimens (0%, 25%, 50%, 75%, and 100% coarse aggregate replacement) showing that increasing the content of *Pachymelania aurita* shells decreased the workability of the concrete. The study recommended using a plasticizer to counteract the reduction in workability [25].

### 2.4. Impact of Oyster Shells on Concrete Strength

Both compressive and flexural strengths were observed to decrease as the percentage of oyster shell replacement increased, with compressive strength dropping by as much as 60% as the oyster shell content increased. Flexural strength also declined, although at a slower rate compared to compressive strength [22]. The partial replacement of fine aggregate with oyster shell was studied, with no significant change in compressive strength as oyster shell substitution increased. The 28-day compressive strength was either equivalent to or greater than that of the control concrete. However, the elastic modulus decreased as the oyster shell substitution ratio increased, due to the lower elastic modulus of oyster shell compared to fine aggregate. The reduction in elastic modulus was approximately 10% for a 20% substitution of fine aggregate with oyster shell [20]. Higher levels of oyster shell substitution were found to negatively affect the long-term strength of concrete. For 10% oyster shell replacement, the long-term strength was nearly identical to that of regular concrete, but at 20% substitution, the rate of strength development slowed, and the overall strength was lower than that of normal concrete [22].

The effects of substituting fine and coarse aggregates with oyster shell in concrete were investigated, with the study observing a decrease in compressive strength as the percentage of oyster shell substitution increased. However, despite this reduction, the results indicated that the concrete still met the necessary standards and could be used for lightweight concrete production [30].

### 2.5. Oyster Shell Characteristics and Chemical Composition

Oyster shells, commonly used as an alternative material in concrete production, exhibit a wide range of colors depending on their geographic origin and environmental factors. Typically, the inner surface of an oyster shell is white or pearl-colored, while the outer shell may range from dark gray to blue, purple, brown, or white, with variations also observed in fresh oysters. These color differences are influenced by factors such as nutrition, temperature, and mineral deposits in seawater, as well as the presence of algae, which may give the shells a spotted appearance.

The primary chemical component of oyster shells is calcium carbonate (CaCO_3_), which makes up more than 90% of the shell’s composition, and is similar to the calcium carbonate used in Portland cement production. Studies have consistently shown that CaCO_3_ constitutes over 95% of the shell material. Oyster shell ash, the powdery residue obtained by calcining oyster shells at high temperatures, has been studied for its potential use in concrete. Table 1 provides a detailed breakdown of the chemical composition of oyster shells, while Table 2 presents the chemical composition of oyster shell ash.

The inclusion of Table 1 and Table 2 in this study is essential for providing a detailed understanding of the chemical composition of oyster shells and oyster shell ash, which are key materials in this research. Table 1 highlights the high percentage of calcium carbonate (CaCO_3_) in oyster shells, confirming their suitability as a potential substitute for traditional cement components in concrete production. Table 2, on the other hand, offers insight into the chemical composition of oyster shell ash, a byproduct of calcining the shells, which may further enhance the material’s applicability in concrete mixtures. By presenting this data, the study aims to underline the relevance of oyster shells as a sustainable material in construction, emphasizing their potential to contribute to both environmental and material efficiency in concrete production.

The reviewed literature highlights the potential of using waste oyster shells as a sustainable alternative in concrete production, with studies investigating their impact on workability, mechanical properties, and durability. While the findings underscore both the benefits and challenges associated with oyster shell incorporation, particularly regarding their chemical composition and substitution ratios, certain gaps remain. Notably, there is limited research on optimizing oyster shell processing techniques to balance mechanical properties and sustainability goals. Additionally, the long-term performance and environmental impact of oyster shell concrete, particularly under varied climatic conditions, require further investigation.

This study aims to address these gaps by evaluating the potential of oyster shells as a fine aggregate replacement in concrete, focusing on optimizing substitution levels and understanding the effects on workability, strength, and durability. By exploring these aspects, this research seeks to contribute to the development of eco-friendly construction materials and advance sustainability in the construction industry.

## 3. Research Methodology

**Figure 1:**
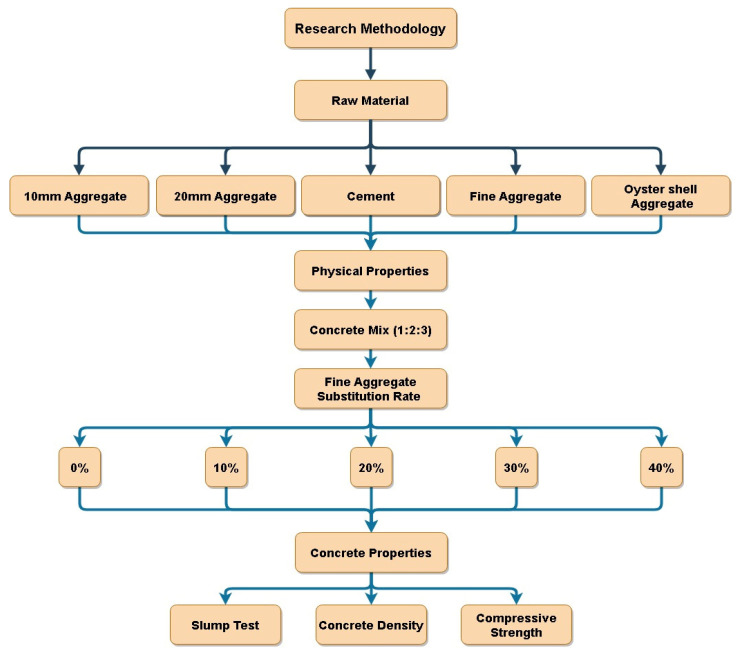
Experimental procedure flowchart.

This section outlines the test procedures and methodologies used to evaluate the fresh and cured properties of concrete containing varying percentages of oyster shell powder as a partial replacement for fine aggregate. Detailed testing procedures for the raw materials used in concrete production, along with the corresponding specifications, are presented. Additionally, the methods employed for mixing, casting, and curing the concrete are discussed comprehensively, including the various tests performed on the cured concrete to determine its mechanical and durability properties. The experimental procedure is illustrated in Figure 1.

The flowchart outlines a research methodology aimed at investigating the effects of oyster shell aggregate as a replacement for fine aggregate in concrete. The study involves using a 1:2:3 concrete mix ratio with varying levels of oyster shell aggregate substitution (0%, 10%, 20%, 30%, and 40%). The resulting concrete specimens are then subjected to tests, including a slump test, density measurement, and compressive strength evaluation, to assess their physical properties. By comparing the results obtained from different substitution levels, this research aims to determine the optimal level of oyster shell aggregate replacement that maximizes desired concrete properties while maintaining acceptable workability.

### 3.1. Mix Design and Sample Preparation

To mix and prepare the concrete, a tilting drum mixer was used. This mechanical device rotates to blend the raw materials into a homogeneous mixture. All ingredients were measured to an accuracy of 1 g. First, the coarse and fine aggregates were placed in the mixer and thoroughly combined. Then, the cement was added until a uniform mixture was achieved. The required amount of water was carefully measured and added, along with a precise dose of water retarder to improve the concrete’s workability. The concrete was mixed in the drum until a fully homogeneous mixture was formed.

The mix proportions used in this study are presented in Table 3. A total of five concrete mixes were prepared, with oyster shell substitutions varying from 0% to 40%, while maintaining a constant water-to-cement ratio of 0.5. The design mix ratio for this study was 1:2:3, meaning 1 part cement, 2 parts fine aggregate, and 3 parts coarse aggregate. The materials were mixed in accordance with ASTM-C192/C192M-19. For each design mix, 12 cylinders with a diameter of 120 mm and height of 240 mm were cast to determine the compressive strength of the concrete. Reusable cast iron molds, compliant with ASTM-C470/C470M-15, were used. Once a homogeneous mixture was achieved, the concrete was poured into the molds in three layers and compacted using a tamping rod.

**Figure 2:**
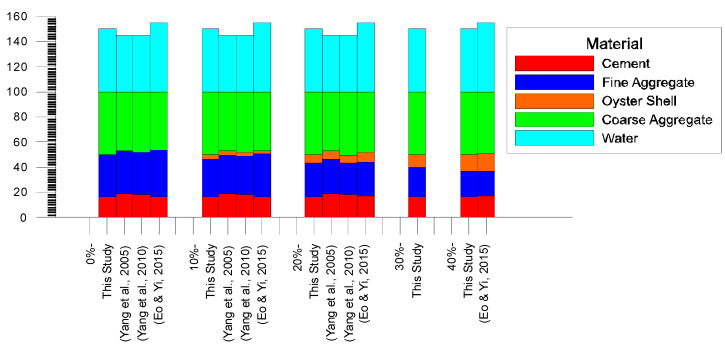
Design mix comparison [20,22,30].

The mix design used in this study was compared with previous studies on replacing oyster shells with fine aggregate (Figure 2). While the percentage of materials used in previous studies is similar to those in this study, the key factor affecting the differences in results is the water-to-cement ratio. The water-to-cement ratio in Yang et al. (2005) and Yang et al. (2010) was 0.45, while in Eo & Yi (2015) it was 0.55 [20,22,30].

### 3.2. Material Experiment

To understand the physical characteristics of the raw material used and the characteristics of the concrete, a series of tests was conducted.

(1) Specific Gravity

In this study, water was used as the standard material for density comparison. The procedure followed the guidelines of ASTM-C127-15 and ASTM-C128-15. Density (*ρ*) is the mass per unit volume of the material under consideration, with the dimensional formula expressed as ML^−3^, where M represents mass, L represents length, and L^3^ represents volume. The equation for specific gravity (SG) can be written as:

(1) $$SG=\rho_{Substance}/\rho_{Water}=M_{Substance}/M_{Water}$$

**Figure 3:**
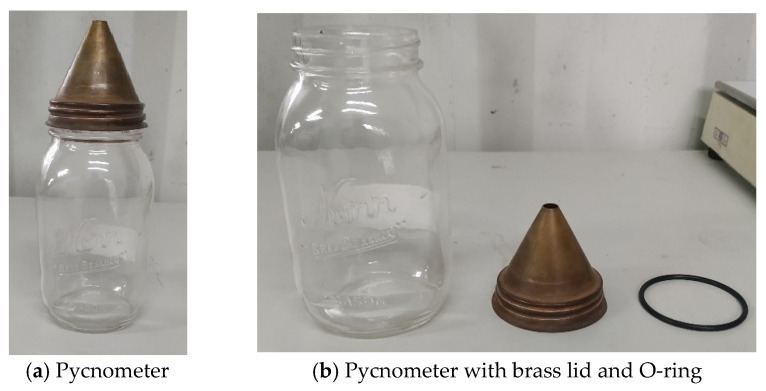
Pycnometer structure.

This study used a pycnometer (Figure 3) to measure the sample. The procedure was as follows:

(1) Before measurements, the sample was dried in an oven at a temperature of 105–110 °C for 24 h.
(2) The weight of the empty pycnometer was measured and recorded as A1.
(3) The oven-dried sample was placed in the pycnometer, and the weight reading was noted as A2.
(4) The pycnometer was then closed with a conical brass top, and distilled water was added through the hole until the pycnometer was filled. This weight was recorded as A3.
(5) The weight of the pycnometer containing only water was measured and noted as A4.

The weight of the sample can be calculated as:

Weight of sample = A2 − A1

The weight of an equal volume of water can be calculated as:

Weight of equal volume of water = (A4 − A1) − (A3 − A2)

Therefore, Equation (1) can be rewritten as:

(2) $$SG=\left(A2-A1\right)/\left(A4-A1\right)-(A3-A2)$$

(2) Absorption of Aggregate

Aggregates consist of solid materials with a small number of voids. The absorption of an aggregate indicates its porosity and the amount of free void space available. Aggregates with higher absorption rates are more porous, making them less durable and weaker. In contrast, aggregates with lower porosity are considered superior, as they have higher strength-bearing capacity. The ASTM standards (ASTM-C127-15; ASTM-C128-15) were followed to test the oyster shell, sand, and coarse aggregate samples.

Absorption is expressed as the percentage of water absorbed by the sample’s pores relative to its dried weight, calculated using the following formula:

(3) $$Absorption\;of\;Sample\left(\%\right)=\frac{\left(W1-W2\right)}{W2}\;\times 100\%$$

where:

W1 = saturated surface dry (SSD) mass; and

W2 = oven-dry mass.

For coarse aggregates, samples were soaked in water for 24 h, then surface dried using a cloth towel to remove excess moisture while leaving inner voids saturated. The SSD mass was recorded as W1. Next, the samples were oven-dried at 105–110 °C for 24 h, and the dry mass was recorded as W2.

For fine aggregates, the sample was soaked in water for 24 h, placed on a flat surface, and blow-dried. To verify if the sample achieved surface dry conditions, an inverted cone was used. The sample was poured into the cone and tamped 25 times to ensure compaction, and to fill the cone to the top. The cone was then carefully lifted. If the sample achieved surface dry conditions, it would slump slightly. If it was too dry, the sample would slump within the cone. In cases where the fine aggregate slumped, water was added, and the cone test repeated. If no slump occurred, the sample had not yet reached surface dry conditions and required further drying. Once the surface dry condition was achieved, the sample was weighed (noted as W1). Finally, the sample was oven-dried at 105–110 °C for 24 h to reach complete dryness, then weighed and recorded as W2.

(3) Sieve Analysis

**Figure 4:**
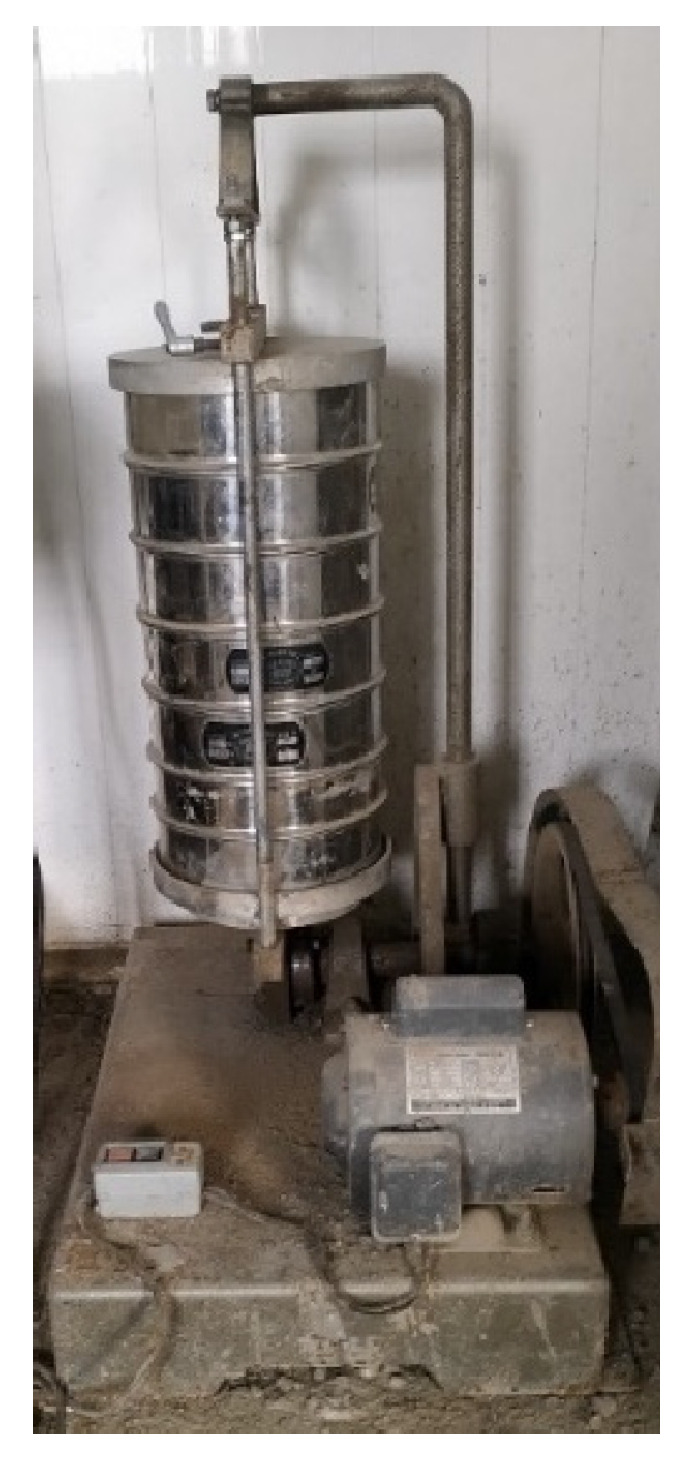
Sieve with sieve shaker.

The particle size distribution and fineness modulus of fine aggregate were determined according to ASTM-C136/C136M-19. A 500 g sample of fine aggregate was used for sieve analysis, and the results were compared with the concrete aggregate specifications outlined in ASTM-C33-18. Multiple sieve analyses were performed on fine aggregate and oyster shells from different batches, with the results averaged. From each batch, a 500 g sample was obtained, and sieves were stacked in sequence: collector pan, 150 μm (No. 100), 300 μm (No. 50), 600 μm (No. 30), 1.18 mm (No. 16), 2.36 mm (No. 8), and 4.75 mm (No. 4), with the collector pan at the bottom and the No. 4 sieve at the top, as shown in Figure 4.

The sample was placed at the top of the sieve stack, covered with a lid, and thoroughly sieved using a mechanical sieve shaker. After sieving, the sample retained on each sieve was carefully weighed and recorded. To understand the particle size distribution, a logarithmic graph of percentage passing versus sieve size was plotted. For calculating the fineness modulus, the cumulative percentage retained on the sieves (150 μm, 300 μm, 600 μm, 1.18 mm, 2.36 mm, and 4.75 mm) was summed and divided by 100. The test results were compared with concrete aggregate specifications (ASTM-C33-18) to ensure compliance.

The uniformity coefficient (Cu) is the ratio of the particle size at 60% finer to the size at 10% finer and is used to measure grain size uniformity. A Cu range of 4 to 6 indicates well-graded material, while a Cu less than 4 suggests poorly graded or uniformly graded material.

The coefficient of curvature (Cc) is calculated by squaring the particle size at 30% finer and dividing by the product of the particle sizes at 10% and 60% finer. For a sample to be classified as well-graded, Cu should fall between 4 and 6, and Cc should be within the range of 1 to 3, while meeting the criteria for Cu. The formulas for Cu and Cc are as follows:

(4) $$Cu=\frac{D_{60}}{D_{10}}$$

(5) $$Cc=\frac{D_{30}^{2}}{{D_{10}\times D}_{60}}$$

where:

D_60_ = particle Size at 60% fineness;

D_30_ = particle Size at 30% fineness; and

D_10_ = particle Size at 10% fineness.

(4) Workability

The workability of concrete refers to how easily it can be mixed, transported, compacted, and finished. While there are several tests to assess concrete flow, the slump test is the most widely used method for measuring workability. The equipment for this test includes a cone, a 500 mm × 500 mm base plate, a 16 mm diameter tamping rod, and a measuring tape. The cone itself has a top diameter of 100 mm, a bottom diameter of 200 mm, and a total height of 300 mm.

To conduct the test, the base plate is placed on a level surface, and the slump cone is positioned in its center. The cone is filled with concrete in three layers, with each layer being tamped 25 times to ensure full compaction. Once the cone is filled with fresh concrete, the top surface is leveled, and the cone is gently lifted to allow the concrete to slump.

The method of measuring slump varies depending on the type of concrete. For normal cement concrete, the procedure follows ASTM-C143/C143M-20. In this case, the slump height is measured, and workability is indicated by the reduction in height after the cone is removed. For self-compacting concrete, ASTM-C1611/C1611M-21 is used. Since self-compacting concrete has high fluidity and low shear strength, it spreads out upon removal of the cone. The workability of self-compacting concrete is assessed by measuring the diameter of the slump flow.

The slump flow measurement is obtained by averaging two perpendicular diameters across the spread concrete. The first measurement, d1, is the largest diameter, while the second measurement, d2, is taken perpendicular to d1. The average of d1 and d2 represents the slump flow, indicating the workability of the concrete mix.

Slump Flow = (d1 + d2)/2(6)

(5) Density Of Concrete

The deadweight of a structure is determined by the density of the concrete, making density an essential property of concrete. Immediately after demolding, each sample was weighed to an accuracy of 0.5 g.

The density was calculated as the ratio of the sample’s mass to its volume, using the following formula:

Density of concrete = Mass of sample/Volume of cylinder

The mass of the sample was measured in kilograms, and the volume in cubic meters. The density is denoted in kg/m^3^.

(6) Compressive Strength

To determine the hardness of concrete, a compressive strength test was conducted using a compression testing machine acquired from Taiwan. The machine’s product model name is CY-6040A15, manufactured by Chun Yen Testing Machines Co., Ltd. This test was performed on cylindrical concrete specimens with a diameter of 120 mm and a height of 240 mm, following the standards outlined in ASTM-C39. The compressive strength of each sample was measured at 3, 7, 28, and 56 days of curing.

During the test, the specimen was centrally positioned between the bearing plates, and a continuous load was applied without any dynamic loading. The load was increased at a constant rate until the specimen failed. The load at the point of failure was recorded as the compressive strength of the test sample.

### 3.3. Material Properties

The materials used in this study were ordinary Portland cement (OPC), fine aggregate, coarse aggregate, and oyster shell. OPC served as the primary binding agent for the concrete. The oyster shell aggregate was sourced from Taiwan Sugar Corp. Waste oyster shells were collected, thoroughly cleaned of organic matter, and crushed to create fine aggregates with an average particle size of 2.55 mm.

All materials for the mix were weighed using a batching scale to measure the precise weights of each component. The test results and properties of all materials are listed below.

(1) Cement

Ordinary Portland cement conforming to CNS-61-R2001 was used for the study. The cement used was manufactured by HT Chunghwa Cement. The specific gravity of the OPC was 3.15 and the initial and final setting times were 100 min and 290 min. The 28-day compressive strength attained by the cement was 45.11 N/mm^2^, as shown in Table 4. The chemical compositions of the cement as per the lab reports were as follows:

Percentage of CaO = 65.50% 

Percentage of SiO_2_ = 20.48% 

Percentage of MgO = 2.06% 

Percentage of Al2O_3_ = 5.93% 

Percentage of SO_3_ = 2.39% 

Percentage of Fe_2_O_3_ = 3.39%

Percentage of Ignition Loss = 0.76%

(2) Oyster Shell

Table 5 presents the specific gravity (S.G.) of oyster shell used in various studies. The oyster shell powder, utilized in this study as a substitute for fine aggregate, had a specific gravity of 2.17 and an absorption rate of 8.71%, as shown in Table 6. Table 7 details the grain size distribution of the oyster shell, confirming its compliance with ASTM C33-18. Notably, the specific gravity of the oyster shell in this study was lower than previously reported values, which may be advantageous for achieving a reduced dead weight in the concrete.

Particle diameter of oyster shell powder at 10% finer (D10) = 0.195 mm

Particle diameter oyster shell powder at 30% finer (D30) = 0.39 mm

Particle diameter oyster shell powder at 60% finer (D60) = 0.74 mm

Fineness modulus = (0.00% + 0.23% + 20.28% + 50.06% + 80.18% + 93.72%)/100 = 2.445

$$\mathrm{Cu}=\frac{D60}{D10}=\frac{0.74}{0.195}=3.795$$

$$\mathrm{Cc}=\frac{{D30}^{2}}{D10\times D60}=\frac{0.39^{2}}{0.195\times 0.74}=1.054$$

**Figure 5:**
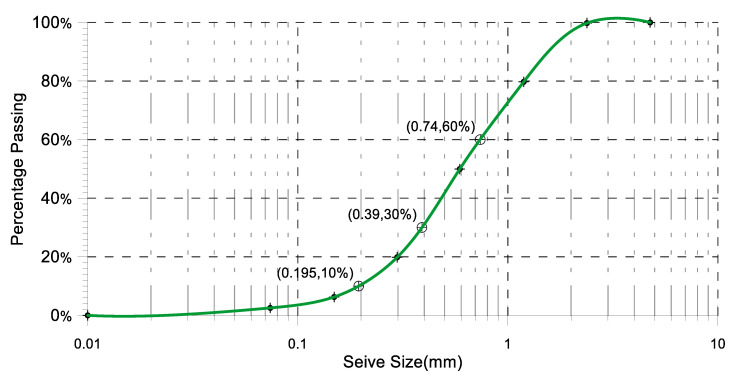
Oyster shell grain size analysis.

Figure 5 illustrates the grain size analysis of the oyster shell. According to ASTM C33-18 (2018), the fineness modulus of fine aggregate should range between 2.3 and 3.1. The fineness modulus of the oyster shell sample fell within this permissible range.

(3) Aggregate

River sand, with a specific gravity of 2.654 and an absorption rate of 2.18%, was utilized as the fine aggregate, as shown in Table 8. The coarse aggregates used in this study were of sizes 10 mm and 20 mm. The specific gravity and absorption for the 10 mm aggregate were estimated to be 2.695 and 1.08%, respectively (Table 9). For the 20 mm aggregate, the specific gravity and absorption were estimated at 2.683 and 0.90%, respectively (Table 10).

**Figure 6:**
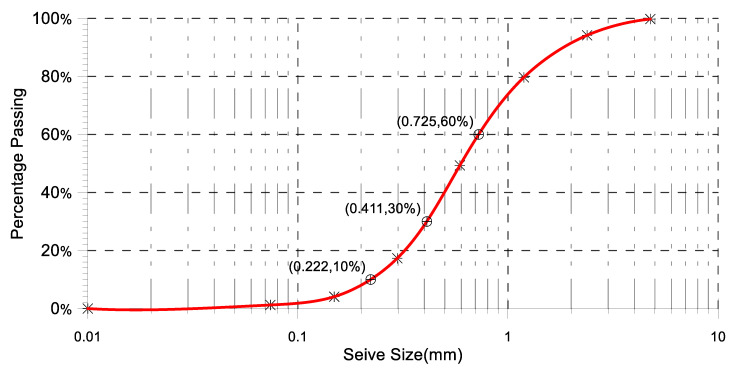
Fine Aggregate grain size analysis.

Table 11 and Figure 6 show the grain size distribution of the fine aggregate employed, as well as its conformance to ASTM-C33-18 (2018).

Particle diameter of OS powder at 10% finer (D10) = 0.222 mm

Particle diameter OS powder at 30% finer (D30) = 0.411 mm

Particle diameter OS powder at 60% finer (D60) = 0.725 mm

Fineness modulus = (0.23% + 5.82% + 20.32% + 50.66% + 82.67% + 95.91%)/100 = 2.556

The fineness modulus of the fine aggregate (ASTM-C33-18, 2018) was between 2.3 and 3.1.

$$\mathrm{Cu}=\frac{D60}{D10}=\frac{0.725}{0.222}=3.266$$

$$\mathrm{Cc}=\frac{{D30}^{2}}{D10\times D60}=\frac{0.411^{2}}{0.222\times 0.725}=1.05$$

(4) Admixture

A water-reducing, high-range admixture was used to enhance the concrete’s workability. This product met the ASTM-C494/C494M-19 standard. Using an admixture at 1.2% to 2.4% of the cementitious material content resulted in a 15% to 30% reduction in water requirement while maintaining the same workability. To maintain consistent workability across all experiments, regardless of the oyster shell content, a fixed dosage of 2% of the admixture, based on the cement weight, was incorporated into each concrete mix.

## 4. Results and Discussion

### 4.1. Workability

**Figure 7:**
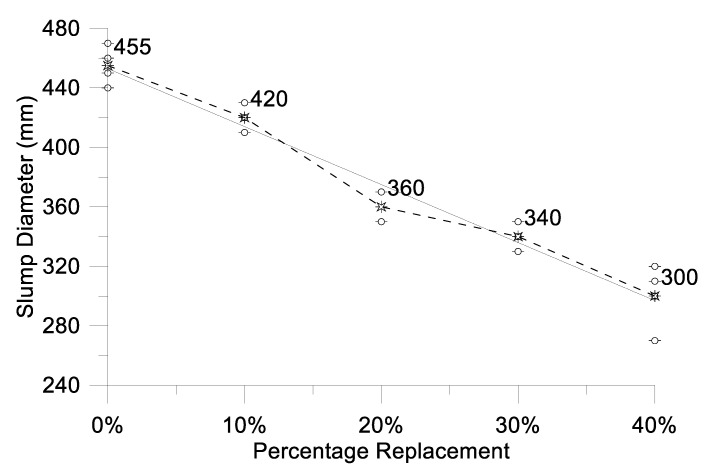
The effect of oyster shell on the concrete slump (The dotted line connecting the mean value of S.D. and the points above and below it is the highest and lowest S.D. respectively.).

Each concrete batch’s workability was assessed prior to casting. Observations show that as the proportion of oyster shell replacement increased, workability decreased. The slump test results, conducted in accordance with ASTM-C1611/C1611M-21, for mixtures incorporating oyster shell substitution are displayed in Table 12 and Figure 7. Analysis of the physical properties of oyster shell powder indicated a higher absorption rate compared to fine aggregate. This higher porosity in oyster shell aggregate led to reduced workability as its content increased in the mix. According to ASTM-C1611/C1611M-21, self-consolidating concrete should have a slump diameter between 480 mm and 680 mm. Given that aggregates (both fine and coarse) constitute 83.33% of the mix, the concrete demonstrated low workability. The recommended slump height for pumped concrete ranges from 50 mm to 100 mm, and in this study, the concrete mix was pumpable due to the inclusion of a high-range water-reducing admixture.

### 4.2. Density of Concrete

**Figure 8:**
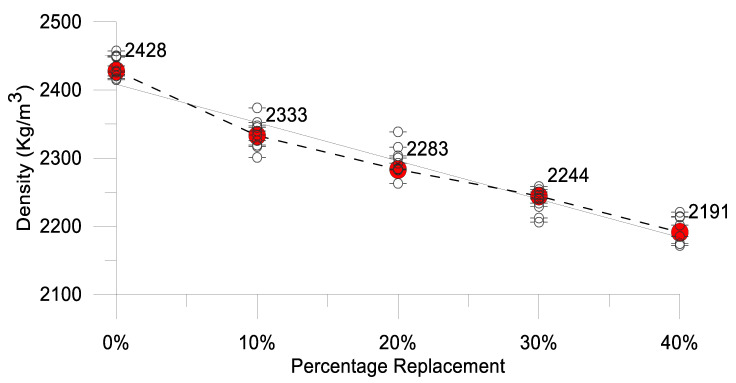
Effect of OS replacement on density of concrete. (The dotted line connects the mean density value to the points above and below it, representing the different density values of each sample).

The density of the concrete decreased as the percentage of oyster shell in the mix increased. With 10%, 20%, 30%, and 40% replacement of fine aggregate with oyster shell, the concrete density decreased by 3.91%, 5.96%, 7.56%, and 9.75%, respectively. Table 13 and Figure 8 provide a visual representation of the density versus percentage of oyster shell replacement.

### 4.3. Compressive Strength

The prepared samples were tested for compressive strength after 3, 7, 28, and 56 days of curing, following ASTM-C39 standards. Each specimen was subjected to a stress rate of 0.25 MPa/s. Table 14 displays a summary of the compressive strength results.

**Figure 9:**
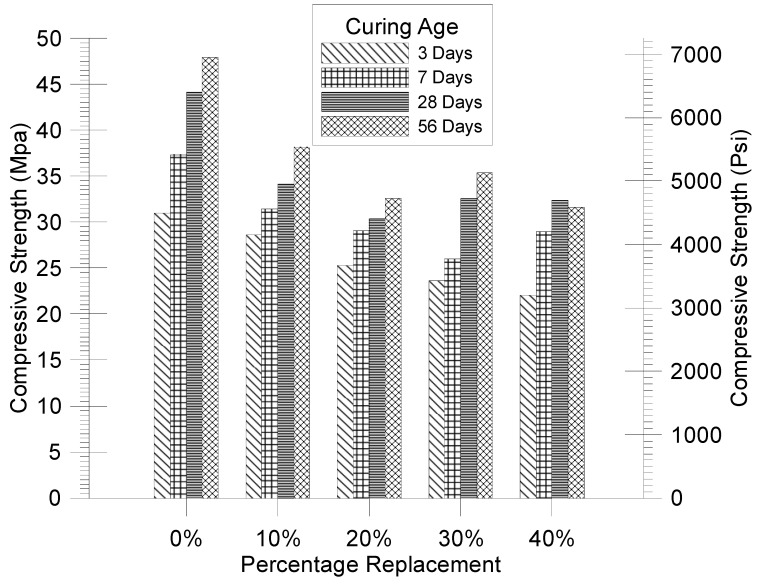
Effect of oyster shell replacement on compressive strength of concrete.

The decline in compressive strength accelerated exponentially with the substitution of fine aggregate. For samples tested on the 28th day, the strength decreased by 22.6%, 31.1%, 26.6%, and 26.7% with 10%, 20%, 30%, and 40% replacement of oyster shell, respectively. On the 56th day, the strength decreased by 20.4%, 32.1%, 26.2%, and 34.1% for the same replacement levels. The development of compressive strength over 3, 7, 28, and 56 days is shown in Figure 9.

For 0%, 10%, and 20% oyster shell replacement, the decrease in compressive strength was linear. However, for 30% and 40% replacement, a similar pattern in strength development was not observed. Notably, the compressive strength of samples with 30% replacement on the 28th and 56th days was higher than that of samples with 20% replacement, even though the 30% replacement samples had a lower density.

In the short term, samples with 40% replacement showed exponential strength development, reaching full maturity by the 28th day, with no further strength gain afterward. Despite being lighter than concrete with 20% replacement, the final compressive strength attained by samples with 40% replacement was similar to that of samples with 20% replacement.

The observed decrease in compressive strength with increasing oyster shell content can be attributed to several key factors. First, oyster shells have a higher porosity and lower specific gravity compared to traditional aggregates. This characteristic leads to a higher water absorption by the oyster shells, which, in turn, increases the water-to-cement ratio in the mix. A higher water-to-cement ratio dilutes the cement paste and reduces its bonding strength, leading to a weaker concrete structure overall. Additionally, the porous nature of oyster shells prevents the formation of a solid bond between the aggregate and the cement matrix, further weakening the concrete.

Furthermore, the increased water demand due to the high absorption of oyster shells results in a reduction in the workability of the concrete mix. As the oyster shell content increases, the slump value decreases, indicating that the mix becomes less workable, which likely contributes to the observed decrease in compressive strength. This suggests that while oyster shells can be used as an aggregate, their higher absorption rate may affect the hydration process, leading to a reduction in strength.

The patterns of compressive strength loss with higher replacement levels of oyster shells were more pronounced at higher percentages, particularly when the oyster shell content exceeded 30%. This is likely because, at higher replacement levels, the increase in internal voids and water absorption became more significant, disrupting the cement hydration process and weakening the concrete matrix. At 40% replacement, the compressive strength showed a steep decline, indicating that the oyster shell aggregate was no longer able to provide adequate structural support to the concrete.

While oyster shells contain calcium carbonate (CaCO_3_), which may have some pozzolanic effects, these effects are minimal compared to the reduction in strength caused by the increased porosity and water absorption. Thus, while the use of oyster shells can be beneficial for certain applications such as lightweight concrete, their use in structural applications requires careful consideration, particularly at higher replacement levels.

In terms of strength behavior, it appears that there is an optimal replacement level of oyster shells—around 30%—where the concrete performs better than at higher replacement percentages. Beyond this threshold, the structural integrity of the concrete is compromised due to the increased porosity and water absorption of the oyster shell aggregate. This finding aligns with similar studies on the use of waste materials, such as seashells and other byproducts, which have also shown a similar trend of strength loss at higher replacement levels.

The inclusion of oyster shell powder as a partial replacement for fine aggregates provides both environmental and material benefits. The primary chemical component, calcium carbonate, interacts with cement during the hydration process. This interaction contributes to the development of strength by enhancing the formation of calcium silicate hydrate (C-S-H) gel. Additionally, the lightweight nature of oyster shells helps reduce the concrete’s overall density, which could lead to a more sustainable material with reduced environmental impact. While the study observed a decrease in compressive strength with higher replacement percentages, the use of oyster shells still provides significant benefits, especially in lightweight concrete applications and waste management. Therefore, oyster shells offer a promising alternative to conventional fine aggregates, improving the sustainability of concrete production.

## 5. Conclusions

Oyster shells, primarily composed of calcium compounds, can be crushed to achieve a grain size distribution similar to that of conventional sand aggregates used in concrete mixes. Comparing the physical characteristics of oyster shells and sand, it is evident that oyster shells are more porous and have a lower specific gravity due to internal voids. This increased porosity in oyster shells is confirmed by the absorption test, showing that oyster shells absorb about four times more water than the fine aggregate used in this study.

The findings of the study are as follows:

(a) Oyster shell aggregate has higher absorption compared to the fine aggregate used in this study. This high absorption increases the water demand of concrete, as oyster shell aggregate has more internal structural pores than sand. Consequently, the workability of the wet concrete decreases, and the slump value decreases as the oyster shell content in the concrete increases.
(b) There is a linear decrease in concrete density with increasing oyster shell content. Because oyster shell aggregate has a lower specific gravity than fine aggregate, the percentage of oyster shell in concrete has an inverse relationship with the concrete’s density.
(c) At a 40% replacement of fine aggregate with oyster shells, a 9.75% decrease in concrete density is observed. The rate of decrease in concrete density is 5.62 kg/cm^3^ per percentage of replacement.
(d) Due to the higher absorption and lower specific gravity of oyster shells compared to the fine aggregates used in this study, oyster shell aggregates are less durable than sand, which contributes to a decrease in compressive strength as oyster shell content increases. However, it is noted that a 30% replacement of fine aggregate performs better in terms of strength than a 20% replacement.
(e) While the durability of concrete decreases with oyster shell content, oyster shells can be effective in developing lightweight concrete and aiding in waste management by repurposing oyster shell powder.

In addition to these findings, this study contributes to sustainable development by promoting the reuse of marine waste, aligning with circular economy principles. Utilizing oyster shells in concrete not only reduces environmental burdens but also offers a viable alternative to natural aggregates, addressing resource scarcity. While the study focuses on mechanical properties, future research could explore methods to enhance durability and workability, such as surface treatments for oyster shell aggregates or the incorporation of admixtures. Furthermore, investigating the performance of oyster shell concrete in various environmental conditions and benchmarking with similar studies could provide a more comprehensive understanding of its potential applications. This research underscores the dual benefits of addressing waste management challenges and advancing sustainable construction practices.

## Figures and Tables

**Table 1:** Chemical ingredient results for oyster shell (%).

Reference	Components (%)							
	CaCO_3_	SiO_2_	MgO	Al_2_O_3_	SrO	P_2_O_5_	Na_2_O	SO_3_
Yoon et al. (2003a) [31]	95.994	0.696	0.649	0.419	0.33	0.204	0.984	0.724
Lee et al. (2008) [32]	95.9	0.69	0.65	0.42	-	0.2	0.98	-
Eo & Yi (2015) [30]	97.244	0.428	0.482	0.449	0.198	0.179	0.539	0.479

**Table 2:** Chemical ingredient results for oyster shell ash (%).

Reference	Component (%)												
	CaO	SiO_2_	MgO	Al_2_O_3_	SrO	P_2_O_5_	Na_2_O	SO_3_	Fe_2_O_3_	K_2_O	TiO_2_	Mn_2_O_3_	* Ig. Loss
Yang et al. (2005) [20]	51.06	2	0.51	0.50	0.09	0.18	0.58	0.60	0.20	0.06	0.02	0.02	44.16
Yoon, et al. (2004) [19]	52.94	0.62	0.78	-	-	0.17	0.93	-	0.32	0.03	0.01	-	44.02
Etuk et al. (2012) [23]	57.95	13.41	0.19	4.95	-	0.01	0.22	0.12	3.80	0.02	0.01	0.01	18.60
Bunyamin & Mukhlis (2020) [33]	51.56	1.60	1.43	0.92	-	-	0.08			0.06		-	42.15

* Includes CO_2_, H_2_O and organic materials lost due to heating.

**Table 3:** Concrete design mix.

OS. Replacement with Sand	W/C Ratio	Mix Percentage				
		Cement	Fine Aggregate	10 mm Aggregate	20 mm Aggregate	OS.
0%	0.5	16.67%	33.33%	25.00%	25.00%	0.00%
10%	0.5	16.67%	30.00%	25.00%	25.00%	3.33%
20%	0.5	16.67%	26.67%	25.00%	25.00%	6.67%
30%	0.5	16.67%	23.33%	25.00%	25.00%	10.00%
40%	0.5	16.67%	20.00%	25.00%	25.00%	13.33%

**Table 4:** Cement compressive strength.

Age of Mortar	Compressive Strength (Kg/cm^2^)	Compressive Strength (MPa)
3 Days	255	25.01
7 Days	362	35.50
28 Days	460	45.11

**Table 5:** Specific gravity (S.G.) of oyster shell used in different studies.

Author	S.G.	Country	Remark
(Eo & Yi, 2015) [30]	2.66	South Korea	Substitution In Concrete
(Yang et al., 2010) [22]	2.48	South Korea	
(Yang et al., 2005) [20]	2.39	South Korea	
This Study	2.17	Taiwan	
(Lertwattanaruk et al., 2012) [24]	2.65	Thailand	Substitution In Mortar
(N. D. Binag, 2016) [28]	3.09	Philippines	
(G.-L. Yoon, Kim, Kim, & Han, 2003b) [31]	2.568	South Korea	
(H. Yoon et al., 2004) [19]	2.41	South Korea	

**Table 6:** Pycnometer reading for OS aggregate.

Description	Sample 1	Sample 2	Sample 3
Weight of empty pycnometer (A1)	435.5 g	435.5 g	435.5 g
Weight of pycnometer + sample (A2)	523.5 g	463.5 g	471.5 g
Weight of pycnometer + sample + water (A3)	1503 g	1470.5 g	1475 g
Weight of pycnometer + water (A4)	1455.5 g	1455.5 g	1455.5 g
Specific gravity	2.173	2.154	2.182
Average specific gravity	2.17		

**Table 7:** Oyster shell grain size distribution.

Sieve No.	Sieve Size (mm)	Percentage Passing	(ASTM-C33-18, 2018)	Percentage Retained
No. 4	4.75	100.00%	95–100%	0.00%
No. 8	2.36	99.77%	80–100%	0.23%
No. 16	1.18	79.72%	50–85%	20.28%
No. 30	0.600	49.94%	25–60%	50.06%
No. 50	0.300	19.82%	5–30%	80.18%
No. 100	0.150	6.28%	0–10%	93.72%
No. 200	0.074	2.54%		97.46%
Collector	Collector	0.00%		100.00%

**Table 8:** Pycnometer reading for fine aggregate.

Description	Sample 1	Sample 2	Sample 3
Weight of empty pycnometer (A1)	435.50 g	435.50 g	435.50 g
Weight of pycnometer + sample (A2)	638.50 g	611.00 g	541.50 g
Weight of pycnometer + sample + water (A3)	1582.00 g	1565.00 g	1521.50 g
Weight of pycnometer + water (A4)	1455.50 g	1455.50 g	1455.50 g
Specific gravity	2.654	2.659	2.650
Average specific gravity	2.654		

**Table 9:** Pycnometer reading for 10 mm aggregate.

Description	Sample 1	Sample 2	Sample 3
Weight of empty pycnometer (A1)	435.5 g	435.5 g	435.5 g
Weight of pycnometer + sample (A2)	599.0 g	612.0 g	639.5 g
Weight of pycnometer + sample + water (A3)	1559.0 g	1566.5 g	1583.0 g
Weight of pycnometer + water (A4)	1455.5 g	1455.5 g	1455.5 g
Specific gravity	2.726	2.695	2.666
Average specific gravity	2.695		

**Table 10:** Pycnometer Reading For 20 mm Aggregate.

Description	Sample 1	Sample 2	Sample 3
Weight of empty pycnometer (A1)	435.50 g	435.50 g	435.50 g
Weight of pycnometer + sample (A2)	638.50 g	611.00 g	541.50 g
Weight of pycnometer + sample + water (A3)	1582.00 g	1566.00 g	1522.16 g
Weight of pycnometer + water (A4)	1455.50 g	1455.50 g	1455.50 g
Specific gravity	2.654	2.700	2.694
Average specific gravity	2.683		

**Table 11:** Fine aggregate grain size distribution.

Sieve No.	Sieve Size (mm)	Percentage Passing	(ASTM-C33-18, 2018)	Percentage Retained
No. 4	4.75	99.77%	95–100%	0.23%
No. 8	2.36	94.18%	80–100%	5.82%
No. 16	1.18	79.68%	50–85%	20.32%
No. 30	0.600	49.34%	25–60%	50.66%
No. 50	0.300	17.33%	5–30%	82.67%
No. 100	0.150	4.09%	0–10%	95.91%
No. 200	0.074	1.23%		98.77%
Collector	Collector	0.00%		100.00%

**Table 12:** Slump diameter (S.D.) of fresh concrete.

Fine Aggregate Replacement	S.D. (d1) (mm)	S.D. (d2) (mm)	S.D. (d1) (mm)	S.D. (d2) (mm)	Mean (mm)
0% Replacement	470	460	450	440	455
10% Replacement	430	420	420	410	420
20% Replacement	370	350	370	350	360
30% Replacement	350	340	340	330	340
40% Replacement	320	310	300	270	300

S.D. = Slump Diameter.

**Table 13:** Density of concrete.

S.No	Density of Concrete (Kg/m^3^)				
	0%	10%	20%	30%	40%
1	2448.11	2333.72	2300.01	2241.43	2214.17
2	2435.22	2300.93	2284.35	2205.88	2174.38
3	2416.80	2344.77	2303.33	2253.77	2184.88
4	2420.48	2373.69	2315.85	2211.96	2214.17
5	2449.59	2318.98	2292.46	2239.96	2174.38
6	2426.01	2325.61	2338.51	2245.48	2184.88
7	2414.96	2347.35	2282.51	2258.19	2171.62
8	2457.32	2352.32	2284.17	2228.91	2220.43
9	2427.85	2339.61	2262.80	2233.88	2213.25
10	2450.18	2316.96	2283.62	2250.09	2201.46
11	2411.27	2317.88	2236.46	2282.69	2141.22
12	2376.27	2322.67	2213.80	2279.01	2197.22
Mean	2427.84	2332.88	2283.16	2244.27	2191.01

**Table 14:** Summary of compressive strength.

Fine Aggregate Replacement	Age	Sample 1	Sample 2	Sample 3	Standard Deviation (σ)	Mean	σ/Mean
0%	3 Days	32.30 MPa	32.41 MPa	28.28 MPa	2.4	31.00 MPa	0.076
	7 Days	37.63 MPa	37.76 MPa	36.64 MPa	0.6	37.34 MPa	0.016
	28 Days	43.64 MPa	46.23 MPa	42.49 MPa	1.9	44.12 MPa	0.043
	56 Days	49.89 MPa	46.61 MPa	47.29 MPa	1.7	47.93 MPa	0.036
10%	3 Days	29.46 MPa	27.79 MPa	28.64 MPa	0.8	28.63 MPa	0.029
	7 Days	32.03 MPa	31.21 MPa	31.03 MPa	0.5	31.42 MPa	0.017
	28 Days	31.08 MPa	35.67 MPa	35.75 MPa	2.7	34.17 MPa	0.078
	56 Days	35.30 MPa	38.93 MPa	40.18 MPa	2.5	38.14 MPa	0.066
20%	3 Days	27.92 MPa	25.35 MPa	22.66 MPa	2.6	25.31 MPa	0.104
	7 Days	29.60 MPa	31.14 MPa	26.36 MPa	2.4	29.03 MPa	0.084
	28 Days	33.88 MPa	29.62 MPa	27.69 MPa	3.2	30.40 MPa	0.104
	56 Days	31.14 MPa	32.90 MPa	33.66 MPa	1.3	32.57 MPa	0.040
30%	3 Days	24.58 MPa	23.92 MPa	22.27 MPa	1.2	23.59 MPa	0.050
	7 Days	28.22 MPa	25.09 MPa	24.74 MPa	1.9	26.02 MPa	0.074
	28 Days	33.93 MPa	32.54 MPa	31.17 MPa	1.4	32.55 MPa	0.042
	56 Days	35.72 MPa	35.45 MPa	34.90 MPa	0.4	35.36 MPa	0.012
40%	3 Days	22.41 MPa	21.80 MPa	22.02 MPa	0.3	22.08 MPa	0.014
	7 Days	29.07 MPa	28.32 MPa	29.52 MPa	0.6	28.97 MPa	0.021
	28 Days	30.14 MPa	34.00 MPa	32.95 MPa	2.0	32.36 MPa	0.062
	56 Days	32.84 MPa	31.73 MPa	30.18 MPa	1.3	31.58 MPa	0.042

## Data Availability

The original contributions presented in this study are included in the article. Further inquiries can be directed to the corresponding author.
